# A system for bioelectronic delivery of treatment directed toward wound healing

**DOI:** 10.1038/s41598-023-41572-w

**Published:** 2023-09-07

**Authors:** Prabhat Baniya, Maryam Tebyani, Narges Asefifeyzabadi, Tiffany Nguyen, Cristian Hernandez, Kan Zhu, Houpu Li, John Selberg, Hao-Chieh Hsieh, Pattawong Pansodtee, Hsin-ya Yang, Cynthia Recendez, Gordon Keller, Wan Shen Hee, Elham Aslankoohi, Roslyn Rivkah Isseroff, Min Zhao, Marcella Gomez, Marco Rolandi, Mircea Teodorescu

**Affiliations:** 1 Department of Electrical and Computer Engineering, University of California Santa Cruz, Santa Cruz, CA 95064 USA; 2 Department of Dermatology, School of Medicine, University of California Davis, Sacramento, CA 95816 USA; 3 Department of Ophthalmology and Vision Science, University of California Davis, Sacramento, CA 95817 USA; 4 Department of Applied Mathematics, University of California Santa Cruz, Santa Cruz, CA 95064 USA; 5 Genomics Institute, University of California Santa Cruz, Santa Cruz, CA 95060 USA

**Keywords:** Electrical and electronic engineering, Drug delivery

## Abstract

The development of wearable bioelectronic systems is a promising approach for optimal delivery of therapeutic treatments. These systems can provide continuous delivery of ions, charged biomolecules, and an electric field for various medical applications. However, rapid prototyping of wearable bioelectronic systems for controlled delivery of specific treatments with a scalable fabrication process is challenging. We present a wearable bioelectronic system comprised of a polydimethylsiloxane (PDMS) device cast in customizable 3D printed molds and a printed circuit board (PCB), which employs commercially available engineering components and tools throughout design and fabrication. The system, featuring solution-filled reservoirs, embedded electrodes, and hydrogel-filled capillary tubing, is assembled modularly. The PDMS and PCB both contain matching through-holes designed to hold metallic contact posts coated with silver epoxy, allowing for mechanical and electrical integration. This assembly scheme allows us to interchange subsystem components, such as various PCB designs and reservoir solutions. We present three PCB designs: a wired version and two battery-powered versions with and without onboard memory. The wired design uses an external voltage controller for device actuation. The battery-powered PCB design uses a microcontroller unit to enable pre-programmed applied voltages and deep sleep mode to prolong battery run time. Finally, the battery-powered PCB with onboard memory is developed to record delivered currents, which enables us to verify treatment dose delivered. To demonstrate the functionality of the platform, the devices are used to deliver H$^+$ in vivo using mouse models and fluoxetine ex vivo using a simulated wound environment. Immunohistochemistry staining shows an improvement of 35.86% in the M1/M2 ratio of H$^+$—treated wounds compared with control wounds, indicating the potential of the platform to improve wound healing.

## Introduction

Wearable bioelectronic devices are a promising approach to personalizing medicine for improved treatment outcomes^1–3^. These devices enable the programmable delivery of drugs for spatiotemporal modulation of biological environments^4^. In comparison, conventional drug delivery methods, such as oral administration, can result in low bioavailability and suboptimal drug dosing^5^. There are many design considerations involved in making bioelectronic devices functional in practice, including wearability, continuous device operation, power management, and wireless communication^6^. Wearable drug delivery platforms should comply with biocompatibility and weight requirements while ensuring accurate dosing. Another consideration is that collecting sufficient data is pivotal in supporting evidence-based practices and for clinical acceptance of treatment strategies employed by bioelectronic devices^7^. Therefore, the manufacturability of bioelectronic devices, specifically the need for scalable prototype production processes, is crucial for producing enough platforms to validate their efficacy. Moreover, many bioelectronic delivery systems require implanting devices to allow for prolonged delivery of ions^8–13^. Wearable wireless devices have been demonstrated for intermittent delivery and signaling with less stringent power requirements^14–16^. Other wearable devices rely on wiring to traditional power supplies^17,18^. Wireless implants have allowed optogenetic experiments in moving mice^19,20^ but require the use of specialized cages for wireless power transfer. We address many of these interdisciplinary challenges through a wearable bioelectronic delivery system. Specifically, the device can sustain continuous delivery of ions, charged biomolecules, and an electric field in vivo and ex vivo. This standalone system can be extended to act as part of a closed-loop wearable drug delivery platform^21^.

**Figure 1:**
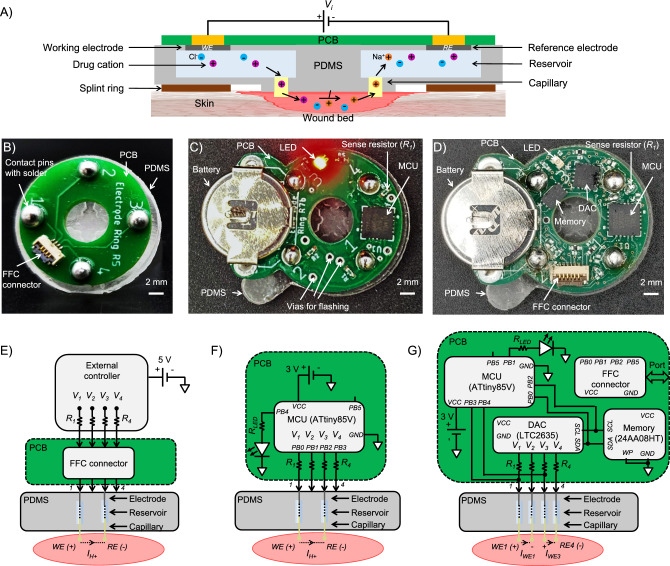
(**A**) The fundamental operating principle of the bioelectronic circuit for the proposed wearable delivery system. (**B**) Wired device for short-duration delivery using an external voltage controller. (**C**) Battery-powered device for long-duration delivery. (**D**) Battery-powered in vivo device with memory for long-duration delivery. (**E**) Circuit diagram of wired PCB showing electrical connection to the PDMS and underlying wound bed. (**F**) Circuit diagram of battery-operated PCB showing electrical connection to the PDMS and wound bed. (**G**) Circuit diagram of battery-operated PCB with memory showing electrical connection to the PDMS and wound bed.

In this paper, we first describe the fundamental operating principle of our proposed bioelectronic delivery system shown in Fig. 1A, which consists of a custom polydimethylsiloxane (PDMS) device that is integrated with a printed circuit board (PCB). We detail the fabrication and integration process of our system, which emphasizes modularity, along with in vivo and ex vivo experimental results for multiple versions of our platform, as shown in Fig. 1B–D, with their respective circuit diagrams in Fig. 1E–G. In particular, modularity in the integration process allows the structure of the PDMS device to remain unchanged while the PCB is updated to achieve varying levels of system functionality. In related work by our group, the devices have been shown to accelerate wound healing in mouse models demonstrated by statistically significant improvement in macrophage M1/M2 ratio and reepithelialization through the delivery of fluoxetine, as detailed by Li et al.^22^ and of an electric field, as detailed by Hernandez et al.^23^.

## Results and discussion

To deliver treatment doses with a biocompatible system, we use PDMS as an intermediary between biological samples, such as a wound bed, and traditional electronic components. We describe the scalable fabrication process and demonstrate the resulting delivery system both in vivo and ex vivo.

Figure 1A shows the bioelectronic circuits of the proposed delivery platform. When a positive voltage $V_i$ is applied between the working electrode (WE) and reference electrode (RE), drug cations (e.g., H$^+$, Flx$^+$, etc.) are pushed through cation-selective hydrogel-filled capillaries from the WE and into the wound bed in exchange for endogenous sodium cations (Na$^+$) at the RE. Figure 1B–D show fabricated prototypes of the wired, battery-powered, and memory-enabled battery-powered system. Each PCB provides four actuation channels whose current can be sensed using resistors, $R_i$, that are in series with the voltages, $V_i$, applied by an external controller or MCU, as shown in Fig. 1E–G. Mechanically, the PCB is ring-shaped to provide a viewport through the PDMS to the biological sample. Electrically, the PCB is connected to the wound bed through electrodes, aqueous reservoirs, and hydrogel-filled capillaries, which are all contained within a custom PDMS body. The electrodes are metal wires whose material can be optimized based on the treatment plan, such as platinum (Pt) wires for H$^+$ delivery or a combination of silver (Ag)/silver chloride (AgCl) wires for Flx$^+$ delivery. The reservoirs can also be filled as required with substances such as H$^+$ or Flx$^+$. Notably, one of the final steps in the fabrication process is filling the PDMS reservoirs, and the voltages applied to the device can be programmed within supply voltage constraints. This provides a modular platform where various treatment plans can be selected by a user or an automated system. The reservoirs are also refillable, provided that the device is unmounted and inactive so it can be handled for refilling via syringe. Finally, the PCBs can be removed from the PDMS device if needed, and reused with a new PDMS device once the posts have been re-coated with silver paste.

This work focuses on expedited wound healing^24^, one application for bioelectronic drug delivery devices. This complex physiological process can be understood as four overlapping phases: hemostasis, inflammation, proliferation, and maturation^25–27^. Wound healing requires the cooperative activity of numerous cell types and information guiding cells to grow and implement tissue morphogenesis to a specific, fully regenerative outcome. To accelerate and improve the wound healing response, a bioelectronic device can provide instructive signals that mimic similar processes in highly regenerative animals. The most important conduits for such signals known to date are the nervous system and migratory immune cells^28–36^. Bioelectronic devices can manipulate the latter of these two central processes by activating macrophages toward reparative functions^37–41^. Macrophages fulfill critical roles during wound healing, spanning from early-stage inflammation to late-stage maturation. Accurately controlling macrophage distribution, migration, and function polarization between the M1 inflammatory phenotype and the M2 anti-inflammatory pro-reparative phenotype in a spatial-temporal manner corresponding to wound status offers a powerful approach to regulated wound healing. In particular, promoting the shift to the M2 pro-reparative phenotype early in the wound healing cycle through H$^+$ delivery and a reduction in pH levels can serve as a significant benchmark of a system’s ability to expedite wound healing.

### Wired bioelectronic system

**Figure 2:**
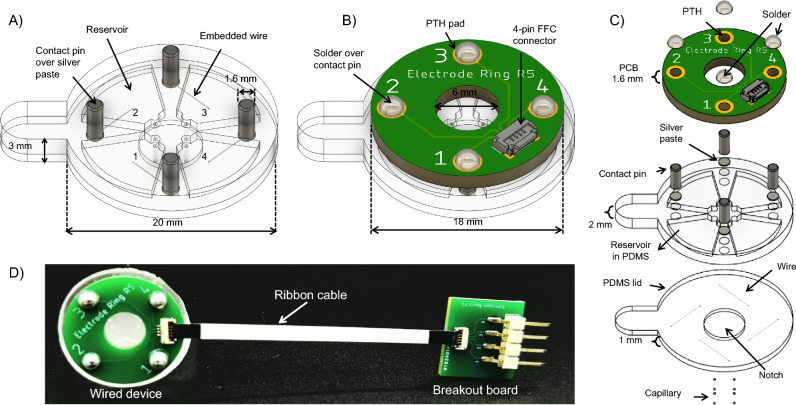
The wired bioelectronic system from Fig. 1B. (**A**) The PDMS device with embedded electrodes and inserted contact posts. (**B**) The complete CAD assembly, with the PCB mounted over the PDMS. (**C**) An exploded view of the wired bioelectronic system device showing individual components/layers. (**D**) The fabricated prototype of the wired device connected to a breakout board via a ribbon cable. The breakout board allows for easier connection to an external voltage controller.

First, we present a wired version of the system (see Fig. 1B), which relies on connecting the PCB to an external voltage controller, based on a design demonstrated by Pansodtee et al.^42^. As a proof of concept, we demonstrate that the platform can achieve ionic delivery when used as a wearable device in splinted, full-thickness wounds on C57B6 mouse models. Figure 2 shows the details of the wired platform, including computer-aided design (CAD) assemblies of the PDMS and PCB subsystems. The PDMS device has 4 reservoirs to hold aqueous source solutions, through-holes to support cylindrical posts for electrical and mechanical interfacing with the PCB, hydrogel-filled capillaries for interfacing with the wound bed, and a protruding notch designed to sit in a recessed wound bed.

**Figure 3:**
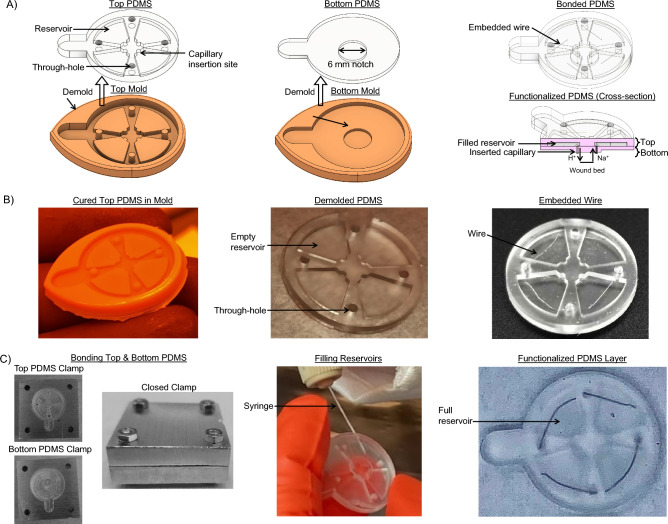
The design and fabrication process of the PDMS device. (**A**) CAD models: top PDMS and mold (left), bottom PDMS and mold (middle), and bonded PDMS (right). (**B**) Top mold filled with PDMS (left), demolded top PDMS piece (middle), and wire insertion (right ). (**C**) Aluminum custom clamps that are used to mechanically secure the two PDMS pieces with electrodes embedded in between. Shown are the PDMS top and bottom pieces in their respective clamps in the two leftmost images. The last three images show the assembled clamp, filling the reservoirs of the bonded PDMS, and the filled PDMS device, respectively.

Figure 3A shows the fabrication process for the PDMS device, which begins with resin 3D printing of two-part molds which are sonicated in IPA, washed off with water, dried with ${\hbox {N}}_{2}$ gas, and finally UV cured. The molds are then filled with recently mixed and degassed PDMS and cured for 48 h in a 60 $^{\circ }$C oven. Next, the two PDMS pieces, referred to as the top and bottom piece, are demolded by running a sharp blade along the edges of the mold, as shown for the top piece in Fig. 3B (left and middle). The top PDMS piece, shown as a CAD model in Fig. 3A (left), contains the reservoirs, through-holes for electronics interfacing, and a capillary insertion site to hold hydrogel-filled capillary tubes which come in contact with the wound surface. The bottom PDMS piece, as shown in Fig. 3A (middle), functions as a lid that seals the exposed face of the reservoirs and features a 0.5 mm tall, 6 mm diameter notch designed to sit in a wound. 4 electrodes are inserted in the top PDMS piece so that one end of the wire is exposed to the reservoir and the other end to a neighboring through-hole, providing an electrical path between them, as shown in Fig. 3B (right). To prevent leakage of solution from the reservoir to the through-hole, we apply a drop of PDMS at the insertion point in the reservoir and allow the drop to cure. To bond the top and bottom PDMS pieces, they are positioned in custom aluminum clamps which are put in an ${\hbox {O}}_{2}$ chamber to activate the exposed PDMS surfaces. The clamp is closed with screws and bolts to ensure mechanical contact and alignment, as shown in Fig. 3C (Bonding Top & Bottom PDMS). Once the devices are inspected to ensure successful bonding and electrode placement, the devices are parylene coated with a thickness of 2.561 $\mu$m to mitigate substance leakage and bubble formation in the reservoirs.

To mechanically interface the PDMS and the PCB body and to provide an electrical bridge from the embedded electrodes to the PCB, 4 low-alloy steel contact posts are inserted in the PDMS through-holes. The contact posts have a 1.6 mm diameter and 4.8 mm length and are located 7 mm from the center of the PDMS device. The post material was selected due to its widespread availability and good solderability, which is comparable to that of copper. The bottom half of the posts are manually coated with conductive silver epoxy paste and inserted into the PDMS through-holes, which make contact with the embedded electrodes. The silver paste in the through-hole hardens and further prevents leakage of reservoir solution into the through-hole. The PCB is designed with 4 plated through-holes (PTHs) that mechanically align with the PDMS through-holes. The PCB is placed flush on the PDMS by guiding the PTHs over the corresponding posts. The protruding top portion of the contact posts is soldered to the pads of the PTHs using a soldering iron, as shown in Fig. 2B.

Finally, the reservoirs are modularly filled with relevant delivery or sensing substances using a syringe, as shown in Fig. 3C (Filling Reservoirs). Hydrogel-filled capillaries are manually inserted in the bottom surface of the PDMS through the protruding 6 mm diameter notch until they reach the reservoirs’ capillary insertion site; this provides a path between the reservoirs and the biological sample that will come in contact with the bottom of the PDMS device. The capillary tubes (inner diameter 100 $\mu$m, outer diameter 375 $\mu$m, 2 - 3 mm tall) are loaded with relevant molecules to provide a path from the reservoirs to the wound bed. The fabricated wired device design weighs 2 g. Also, the PCB houses a side-entry, 0.5 mm pitch, flexible flat cable (FFC) connector. The wired device is connected to an external voltage controlled via a breakout board and ribbon cable, as shown in Fig. 2D. The time taken for completion of each fabrication step to produce a batch of 24 devices are listed in Table 1. We have demonstrated the biocompatibility of the portion of our devices that are in contact with the wound. The experimental design and results of biocompatibility testing are listed in Supplementary Table S1. See supplementary information for further explanation of the biocompatibility experiments conducted.

**Table 1:** Device fabrication steps and associated completion times for a batch of 24 devices.

Step	Fabrication process	Active time	Passive time
1	3D printing molds	–	10 h
2	Cleaning molds	1 h	2 h
3	UV cure molds	–	40 min
4	Pour PDMS and degas	20 min	40 min
5	Bake PDMS	–	48 h
6	Demold PDMS	32 min	–
7	Clean PDMS	15 min	–
8	Wire insertion	1 h	20 min
9	PDMS bonding	1 h 5 min	20 min
10	Parylene-C coating	2 h	10 min
11	Treat capillaries	1 h	26 h
12	Fill capillaries w/ hydrogel	30 min	–
13	UV crosslink hydrogel	5 min	30 min
14	Capillary insertion	1 h 30 min	–
15	Reservoir filling	3 h	–
16	PCB integration (silver paste + solder posts)	2 h	30 min
	**Total batch production time**	14 h 17 min	89 h 10 min
	**Total device production time**	36 min	3 h 43 min

The active time to produce one device is estimated as 36 min.

In summary, hydrogel-filled capillaries connect the wound bed to the reservoirs, which connect to contact posts soldered to the PCB PTHs via embedded electrodes, representing a path from the PCB to the wound bed. The fabrication of the PDMS device body and integration with the PCB provides a reliable and scalable process to produce the bioelectronic systems.

#### H$^+$ delivery

We perform H$^+$ delivery ex vivo and in vivo to demonstrate the device’s functionality. The PDMS device is prepared for H$^+$ delivery by embedding 4 Pt electrodes in the top PDMS piece before the bonding procedure, and the wired PCB design is soldered onto the contact posts. Pt electrodes were chosen for H$^+$ delivery as they gave higher current responses than Ag. Reservoirs 1 and 3 are filled with 0.5 M HCl while reservoirs 2 and 4 are kept empty (see Fig. 2A), and all 4 capillary tubes are loaded with H$^+$ (see Fig. 2C). 0.5 M HCl concentration was chosen to provide an environment abundant with H$^+$ ions but a lower concentration of around 0.1 M HCl can be used to minimize tissue damage in the event of leakage.

Actuating the device consists of applying a voltage $$V_i$$ at channel $$i = 1$$ and $$i = 3$$ across the two Pt electrodes (WE and RE) in the filled reservoirs 1 and 3, respectively, which drives H$$^+$$ through the cation-selective 2-acrylamido-2-methyl-1-propanesulfonic acid (AMPSA)-polyethylene glycol diacrylate (PEGDA) polyanion hydrogel in the device capillaries (see Fig. 1E). Polyanion hydrogel is commonly considered as a selective barrier that only allows cations to pass while blocking the anions^12,43^. The applied voltage $$V_i$$ at each channel for H$$^+$$ delivery is listed in Table 2. The 0.5 M HCl solution in the PDMS reservoirs is the source of H$$^+$$ ions for delivery. For a positive $$V_i$$, H$$^+$$ is pushed from the reservoir containing the WE to the wound due to the Coulomb force. To maintain charge balance, endogenous cations, primarily Na$$^+$$^44–46^, present in the wound are pulled into the reservoir containing the RE, as depicted in Fig. 3A (right). The exchange of endogenous cations is necessary for electrophoretic delivery of ions and charged biomolecules^47,48^ as it establishes current flow, and hence, mechanism for charge transfer. Further research is needed on whether endogenous cation exchange has a direct effect on the wound healing process. The counter-ion and endogenous anions, primarily Cl$$^-$$, also contribute to the total current $$I_{H^+}$$ between the WE and RE. Thus, H$$^+$$ can be delivered to a target biological sample, and an approximate delivered concentration can be estimated from $$I_{H^+}$$. At first, the wired devices are tested for 4 days ex vivo on the surface of chicken breast as it shows similar current profile and levels as that of a mouse wound (see Supplementary Fig. S1 online). For the wired device, an external voltage controller is used to determine if the current levels are high enough for H$$^+$$ delivery (see Supplementary Figs. S1 and S2 online). More importantly, the current output of the devices, and therefore, the hydrogel-filled capillaries, does not degrade over a period of 4 days, as shown in Supplementary Fig. S1 online. As depicted in Fig. 1E, the current in each channel *i* is measured by the controller using 4 current sense resistors $$R_i$$ = 1 k$$\varOmega \pm$$0.1%.

**Table 2:** Applied voltage $V_i$ at the four channels for 8 to 10 min H$^+$ delivery with the wired bioelectronic system.

Channel	Ch. 1 (WE)	Ch. 2	Ch. 3 (RE)	Ch. 4
Applied voltage ${{\varvec{V}}}_{{\varvec{i}}}$	1.5 to 2 V	NC	0 V	NC

Reservoirs 2 and 4 are empty and not connected (NC) to the wound bed.

After validating the platform’s ability to deliver H$^+$ ex vivo, the platform was tested in a four-day in vivo experiment. Devices were affixed to mouse wounds and successfully actuated for H$^+$ delivery, remaining intact and functional throughout the length of the experiment. In the experiment, three mice were used, each with two circular 6 mm splinted excisional wounds on either side of the spine. One wound was used for control testing, consisting of a non-functionalized PDMS device (no PCB, unfilled reservoirs, unloaded capillaries). The other wound was used for device testing, consisting of the entire device assembly configured as specified for the wired device design test.

**Figure 4:**
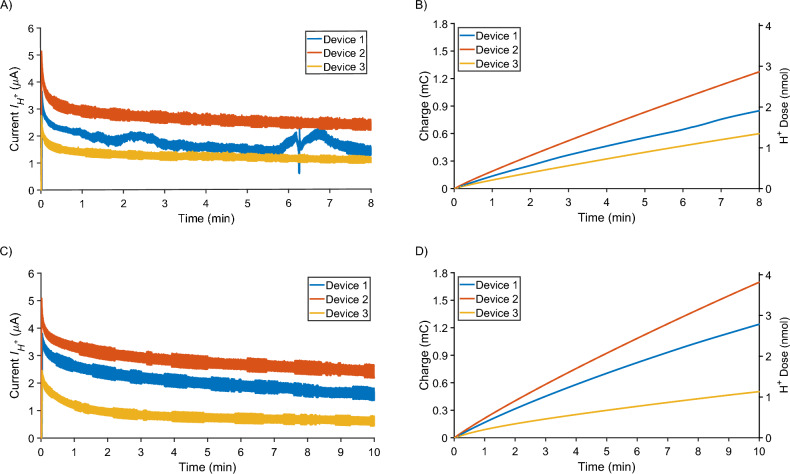
Wired system actuation results from the in vivo experiment on Day 0 and Day 1. (**A**) Current responses $I_{H^+}$ on Day 0, where $V_i = 1.5$ V was applied across WE and RE on each device for 8 min (shorter duration to allow time for surgery). (**B**) Total charge and accumulated dose on Day 0. (**C**) Current responses $I_{H^+}$ on Day 1, where $V_i = 2$ V was applied across WE and RE on each device for 10 min (longer duration since surgery was not required). (**D**) Total charge and accumulated dose on Day 1.

On Day 0 of the experiment, wounds were surgically generated, and images of all wounds were captured. Next, the experimental devices were actuated for an 8 min period using the external voltage controller wired to the PCB of the bandage, as illustrated in Fig. 1E and Supplementary Fig. S2 online. The actuation duration was limited to 8 min by the allowable anesthesia time for the mice. After the actuation period, devices were secured to the mouse wounds using Tegaderm. On Day 1, mice were re-anesthetized, and devices were actuated for 10 min, with an additional 2 minutes allowed because the surgery was only required on Day 0. The resultant current responses $I_{H^+}$, total charge, and accumulated dose from the device actuation across Day 0 and Day 1 are shown in Fig. 4.

The total charge *Q* in Coulomb (C) at time *t* can be calculated by integrating current response $I_{H^+}$ over a time period of $(t - t_{start})$ as

1 $$\begin{aligned} Q (t)= \int _{t_{start}}^{t} I_{H^+}(\tau ) d\tau \qquad \text {(C)} \end{aligned}$$

The accumulated dose *D*(*t*) in mol can be calculated as

2 $$\begin{aligned} D (t) = \frac{\eta Q (t)}{F} \qquad \text {(mol)} \end{aligned}$$

where $\eta$ is the average H$^+$ delivery efficiency and $F = 96485.3321$ C/mol is the Faraday’s constant.

The endogenous Na$^+$ and Cl$^-$ ions compete with H$^+$ ions to carry the current^46^. The H$^+$ delivery efficiency was determined to be $\eta = 21.7\%$ across all devices using the method utilized by Dechiraju et al.^49^. Based on equation (1), the current responses $I_{H^+}$ in Fig. 4A and C are integrated over time in MATLAB using the cumulative trapezoidal method. Then, using Eq. (2), we found that around 1.91, 2.86, and 1.35 nmol of H$^+$ were delivered by the three devices on Day 0, and 2.79, 3.82, and 1.13 nmol of H$^+$ were delivered on Day 1, as shown in Fig. 4B, D.

Devices were not actuated after Day 1, as lowering pH past the inflammation stage of wound healing may impede the re-epithelialization of the wound^50^. In particular, macrophages have been studied through conditional deletion during different stages of amphibian^51^, and mouse healing has revealed stage-specific functionalities. Deletion early in the wound trajectory diminishes granulation tissue and myofibroblast formation, while depletion during the mid-stage of healing destabilizes the existing vasculature and impairs epithelialization.

On Day 2 of the experiment, devices were visually inspected to ensure no tampering from the mice had occurred. On Day 3, wound images were again captured, the mice were sacrificed, and the wound tissue was harvested for IHC staining. The accumulated dose delivered by the devices for each day is summarized in Table 3.

**Table 3:** H$^+$ dose delivered to the wounds by the wired system on Day 0 and Day 1, along with corresponding changes in local pH.

Day	Device	H$^+$ Dose *D* (nmol)	Final pH
Day 0	1	1.91	6.99
	2	2.86	6.99
	3	1.35	6.99
Day 1	1	2.79	6.99
	2	3.82	6.98
	3	1.13	6.99

We calculated the pH change in a localized delivery area of 5 $\mu$L, after which H$^+$ is assumed to diffuse throughout the remainder of the wound bed. We also assumed the buffering capacity of wound fluid to be near that of blood at physiologically relevant pH levels, 38.5 mEq/L/pH^52^. Ultimately, testing our device for H$^+$ delivery can be evaluated by the reduction of the local pH. pH estimates are shown in Table 3, assuming an initial wound pH of 7^43^.

### Battery-powered bioelectronic system

Upon validation of device functionality using the wired PCB, we increased the PCB’s complexity by integrating a battery and an onboard microcontroller unit (MCU) for remote, continuous delivery. This modification, detailed in Fig. 1C, facilitates extended actuation periods and allows for increased cumulative treatment doses. An in vivo experiment is performed with this system, and immunohistochemistry (IHC) staining is used to examine the resulting macrophage populations. We demonstrate that this system successfully activates macrophages vital to wound healing^19,20,53–55^.

**Figure 5:**
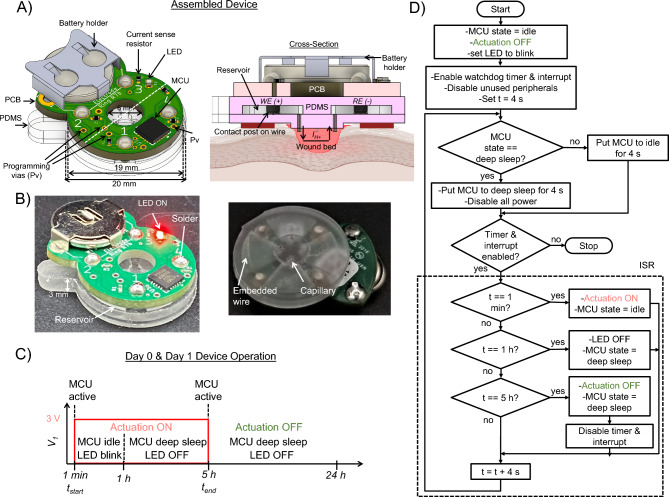
Programmable battery-powered system. (**A**) CAD models of integrated PCB-PDMS device with MCU. Perspective view (left) and cross-section view device on a wounded skin (right) [Created with BioRender.com]. (**B**) Fabricated prototypes. Perspective (left) and isometric bottom view (right). (**C**) Timing diagram of the applied voltage $V_1$ waveform. (**D**) Flowchart of control program execution for H$^+$ delivery.

A wireless PCB is more practical as it allows for unrestricted mouse movement during device actuation. Figure 5A, B show the CAD model and fabricated prototype of the battery-operated device, respectively. The battery-powered PCB consists of a small, low-power MCU [Microchip ATtiny85V QFN package]^56^ that can be pre-programmed for timed wound treatments; the controller automatically turns off the electrical actuation signal after a specified duration. This wireless PCB resembles a figure-eight shape; the main ring has an outer diameter of 19 mm, and the semi-circular extension holds a 12 mm coin-cell 3 V battery. The footprint of the PCB is 19 mm $\times$ 25 mm with a height of 1.6 mm. The PDMS device and integration method remain as shown in Fig. 5A, which significantly reduced the development time for updating from a wired to a wireless device. Fabrication steps and production times remain the same, as listed in Table 1.

The assembled battery-powered device weighs 3.6 g. As depicted in Fig. 1F, the four output pins of the MCU are individually connected through the current sense resistors, $R_i$ = 10 k$\varOmega \pm$0.1%, to four electrodes in the PDMS. Each electrode/channel *i* can be pre-programmed to be either the WE (by setting $V_i = 3$ V) or the RE (by setting $V_i = 0$ V) for a specified duration. An ultra-low current LED (2 mA forward current) is used to indicate whether the actuation is ON or OFF. When the actuation is ON, the applied voltages at channel 1 (WE) and 3 (RE) are set to $V_1 = 3$ V and $V_3 = 0$ V, respectively, to provide current $I_{H^+}$ to the wound bed, and the LED blinks at a low frequency and low duty cycle. During this period, the MCU is kept in idle mode to reduce the power consumption and prolong battery run time. When the actuation is OFF, the applied voltages are set to $V_1 = V_3 = 0$ V, the LED is turned off, $I_{H^+} \approx 0$ $\mu$A, and the MCU is put into a deep-sleep mode. The MCU is momentarily active (draws most current) only when switching between actuation ON and OFF, as depicted in the timing diagram shown in Fig. 5C. The measured power consumption of the battery-powered devices is only 40 $\mu$W (under a typical actuation/load current of $I_{H^+}$ = 10 $\mu$A) when the MCU is put into deep-sleep mode. Power consumption is obtained by multiplying the battery supply voltage and current, both measured using a high-precision multimeter. The deep sleep mode is utilized for around 80% of the actuation ON time. The device is initially switched to idle mode during the first hour to make the LED blink. During this period, the average power consumption is 2.2 mW. Notably, when the actuation is OFF, the power consumption is further reduced to 0.45 $\mu$W since there is no load current and the watchdog timer is disabled. Table 4 shows the comparison of power consumption and actuation parameters of this system with similar wearable, standalone bioelectronic drug delivery and electrical stimulation systems found in the literature. The deep sleep power management approach allows for up to 3 days of continuous operation of the battery-powered bioelectronic device. The applied voltage $V_i$ at each channel for H$^+$ delivery is summarized in Table 5. To keep the total current $I_{H^+}$ levels low, only reservoirs 1 and 3 are filled (see Fig. 2A) and the other two are kept empty to avoid connecting them electrically to the wound bed. An initial 1 min setup period (actuation OFF) was pre-programmed to follow battery insertion and allow enough time to establish voltage measurements across the current sense resistors with a multimeter.

**Table 4:** The battery-powered and memory enabled battery-powered bioelectronic systems in comparison with other wearable bioelectronic systems.

Device	Power consumption ($\mu$W)	Applied voltage (V)	Actuation current ($\mu$A)	Actuation duration (per day)	Frequency
Battery-powered bioelectronic system	40	3	0–20	5–7 h	DC
Memory enabled battery-powered bioelectronic system	1836	3	0–20	5–7 h	DC
Stretchable, wireless bioelectronic system^1^	–	1	1000	10–20 min	DC
Wireless smart wound dressing^14^	30	−0.5	–	90 s	DC
Triboelectric nanogenerator^16^	–	0.2–2.2	–	24 h	0.5–1.83 Hz
Piezoelectric nanogenerator^57^	–	0.1–+0.5	–	24 h	1 Hz
Nanofluidic membrane^58^	45	−3, −1.5	–	8 h	DC
Wireless, closed-loop, smart bandage^59^	–	0–2	–	6 h	13.56 MHz (rectified AC)
Wireless bioresorbable system^60^	–	0.1–0.3	–	1 h	20 Hz (200 $\mu$s pulse)
Self-powered, on-demand drug delivery system^61^	–	> −0.8	–	15-20 min	–

**Table 5:** Applied voltage $V_i$ at the four channels for 5 h H$^+$ delivery with the battery-powered system.

Channel	Ch. 1 (WE)	Ch. 2	Ch. 3 (RE)	Ch. 4
Applied voltage ${{\varvec{V}}}_{{\varvec{i}}}$	3 V	NC	0 V	NC

Figure 5D shows the algorithmic flowchart of the control program execution. The control program was written in C++ using the Arduino IDE environment. The internal watchdog timer of ATtiny85V is used to keep track of the actuation duration. The tracking is done by an interrupt service routine (ISR) which is executed every 4 s when the timer times out. Each ISR execution increments a count variable, so each count represents 4 s. Once the accumulated count (i.e., time) reaches the specified actuation duration, the actuation is turned OFF.

The MCU on the PCB is flashed with Arduino Uno as an in-system programmer (ISP) by connecting the Uno’s four serial peripheral interface (SPI) pins, 5V, and GND pins to the programming vias (see Fig. 5A and Supplementary Fig. S3) using 22 AWG wires. To verify the timing of signals generated by the MCU, a control program was written to produce a logic high output at channel 1 (WE) and a logic low output at channel 3 (RE) for 60 s. Further, to verify that the applied voltage is capable of driving the anticipated electrical load of the system, a simulated load (1.2 M$\varOmega$ resistor representative of combined capillaries, reservoirs, and solution/wound resistance) was connected across channels 1 (WE) and 3 (RE) of the PCB, as shown in Supplementary Fig. S4.

During the actuation ON time, a digital oscilloscope was used to measure a voltage difference of 2.8 V (near the battery voltage) between WE and RE, corresponding to a current of 2.3 $\upmu$A, as shown in Supplementary Fig. S4 online. The measured actuation ON and OFF duration is 56 s which is close to the set pulse width values of 60 s with a 50% duty cycle.

#### H$^+$ delivery

We perform bioelectronic delivery of H$^+$ to test the battery-powered system. We prepare the PDMS as previously, using Pt electrodes, 0.5 M HCl filled reservoirs, and H$^+$ loaded capillaries. The voltage waveform, depicted in Fig. 5C, is initiated by inserting the battery into the PCB battery holder. The MCU on the PCB was programmed to apply $V_i = 3$ V across the WE and RE for 5 h to drive H$^+$ from the reservoir containing the WE. The ion transport process is the same as the wired system, where H$^+$ is delivered through the device capillary and into the wound bed. Initial current measurements are made using a multimeter. At first, the devices are tested ex vivo on chicken breast, and voltages across the current sense resistors are measured (see Supplementary Fig. S5 online).

As with the previous experiment, a four-day in vivo experiment was conducted with this device to deliver H$^+$. The devices were actuated for a 5 h period on Day 0 and 1 to reduce the local wound pH to 6. Again, no actuation was conducted on Day 2 and 3 so as to reduce inflammation time but not compromise re-epithelialization due to later decreases in pH^50^. Commercial 0.9% saline solution used for infusion and wound wash has a pH of around 5.5, sometimes even reaching a pH value as low as 4.6^62^. The devices will not induce excessive acidosis when considering that local wound pH does not get below 6 after the 5 h actuation. Six mice were used for the experiment, each with one circular 6 mm splinted excisional wound on a side of their spine. In accordance with weight restrictions, there was only one wound created per mouse due to the slightly higher weight of the battery-powered device. Two mouse wounds were assigned as controls, with each control consisting of an assembled device with empty reservoirs. The remaining four mouse wounds were used for testing the actuated devices. The currents $I_{H^+}$ measured when the four devices were placed and actuated on mouse wounds are summarized in Table 6.

**Table 6:** Current $I_{H^+}$ measured on channel 1 of the battery-powered system using a multimeter (across the $R_1$ sense resistor) at the start of 5 h actuation for H$^+$ delivery in mice on Day 0.

Device	Current ${I_{H^+}}$ (${\mu }$A)
1	10
2	20
3	17
4	14

We evaluate the effectiveness of the battery-powered bioelectronic system in vivo through IHC staining to determine the presence and distribution of macrophages within tissue samples. Supplementary Fig. S6 presents IHC staining results for the two control and the four H$^+$ – treated mice, which were used to calculate the ratio of M1 and M2 macrophages within respective samples. The staining results show that, on average, the M1/M2 ratio of the H$^+$ – treated wounds is 35.86% lower compared to the control wound. The lower M1/M2 ratio indicates that, on average, in the treated wounds, there is a relative increase of M2 macrophages, which is known to promote wound healing through tissue repair and regeneration^41,63^. See supplementary information for further explanation.

### Memory enabled battery-powered bioelectronic system

Building upon the proof of concept for the battery-powered PCB with an MCU, we further advanced the PCB design to include onboard electrically erasable and programmable read-only memory (EEPROM). This device (see Fig. 1D) is capable of recording treatment delivery as a function of time. This is demonstrated by recording the delivery of the fluoxetine cation (Flx$^+$), also associated with improved wound healing^64–66^. The current recordings from ex vivo experiments validate that this system can continuously deliver a charged biomolecule (fluoxetine) for up to 7 h.

**Figure 6:**
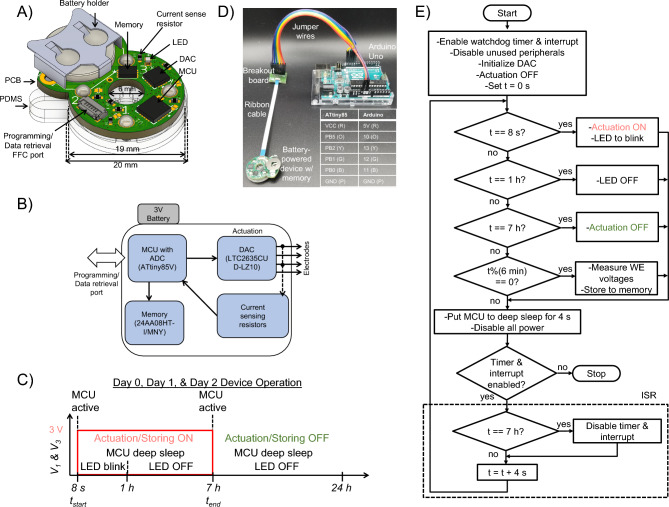
Programmable battery-powered bioelectronic system with memory. (**A**) CAD model showing the integrated PCB-PDMS device. (**B**) Block diagram of the PCB electronics. (**C**) Timing diagram of the applied voltages $V_1$ (channel 1) and $V_3$ (channel 3). (**D**) The programming process for a fabricated prototype. The device is connected to an Arduino Uno via a breakout board. (**E**) Flowchart of control program execution for Flx$^+$ delivery.

Figure 6A, B shows the memory-enabled battery-powered design with the onboard EEPROM chip (Microchip Technology 24AA08HT-I/MNY) and digital-to-analog converter (DAC) [Analog Devices LTC2635CUD-LZ10]. The fabricated device weighs 3.7 g. See Table 1 for fabrication steps and production times, which remain the same for this system as well. As depicted in the circuit diagram shown in Fig. 1G, the I^2^C clock (SCL) and data (SDA) pins of the onboard MCU are connected to the respective pins of the DAC and EEPROM chips for communication. The MCU is kept in an active mode during sampling and data storing but put into deep-sleep mode at other times to conserve battery, as indicated in the timing diagram shown in Fig. 6C. The memory enabled battery-powered system consumes 1.8 mW when the MCU is in deep sleep, under a typical actuation (load) current of $I_{WE}$ = 10 $\mu$A. During the first hour when the LED is blinking, the average power consumption is 2.7 mW. Table 4 compares this device with other wearable devices in the literature. Jumper wires and ribbon cables are used to connect the Arduino (as an ISP) through a breakout board to the controller, as depicted by the fabricated prototype, breakout board, and cable assembly in Fig. 6D shows.

Table 7 lists the main controller specifications. The MCU is flashed with a control program through a 6-pin FFC connector connected to an Arduino Uno’s four SPI (10, 11, 12, 13), 5V, and GND pins. The Arduino is also used to retrieve the data from the onboard memory of the controller via its I^2^C bus (SCL, SDA), 5V, and GND pins, using the same ribbon cable and breakout board assembly with the PB2 (Y) and PB0 (B) jumper wires connected to the SCL and SDA pins, respectively, of the Arduino. The MCU is flashed with a blank program before reading the EEPROM to avoid I^2^C bus conflicts with the Arduino during the retrieval. The battery provides the supply voltage $V_{CC}$ = 3 V. The DAC has a 10-bit resolution with four channels and can provide a variable applied voltage $V_i$ in the range from 0 to 3 V at its four outputs that are individually connected to the four electrodes through the current sense resistors, $R_i$ = 10 k$\varOmega$. As shown in Fig. 1G, two of these electrodes are connected to the built-in ADC of the MCU to enable sensing of WE voltages $V_{WE1}$ and $V_{WE3}$ and currents $I_{WE1}$ and $I_{WE3}$ at the channels $i = 1$ and $i = 3$, respectively. The ADC has a 10-bit resolution, translating to a 3 mV resolution for voltage and 0.3 $\mu$A for current measurements. At every 6 min recording interval, the ADC samples the WE voltages and converts them into 10-bit integers for channels $i = 1$ and $i = 3$. A burst of sixteen samples spaced 67 ms apart is taken for each channel of interest. The MCU then finds the average of these samples to reduce noise. The MCU stores the point-averaged 10-bit integer code $DD_i$ on the EEPROM chip via the I^2^C bus for channels $i = 1$ and $i = 3$. The average WE voltage at channels $i = 1$ and $i = 3$ can be found as

3 $$\begin{aligned} V_{WEi}=\frac{DD_i}{1024} V_{CC} \end{aligned}$$

WE voltage from equation (3) is converted to WE current value for channels $i = 1$ and $i = 3$ as

4 $$\begin{aligned} I_{WEi} = \frac{V_{i}-V_{WEi}}{R_{i}} \end{aligned}$$

**Table 7:** Specifications of battery-powered PCB with memory.

Parameter	Specifications
Channels	4-actuation (DAC), 2-sensing (ADC)
Applied voltage $V_i$	0 to 3 V
WE current $I_{WEi}$	0 to 300 $\mu$A
Sensing resolution	10-bit ADC (3 mV, 0.3 $\mu$A)
Averaging factor	16
Sampling period	67 ms
Power management	Active/deep sleep
Typical delivery duration	7 h per day (up to 3 d)
Memory (EEPROM) size	8 Kbit (4 $\times$ 2Kbit)
Current sensing resistors	10 k$\varOmega \pm$0.1%

Additionally, an ultra-low current LED is used to visually indicate the tentative amount of total WE current $I_{WE} = I_{WE1} + I_{WE3}$ delivered. Between every 4 s interval, the LED blinks 0 to 5 times to indicate total current $I_{WE}$ in the range 0–1, 1–2, 2–3, 3–4, 4–5, and $\ge$ 5 $\mu$A, respectively. The control program execution for fluoxetine delivery is shown in Fig. 6E.

#### Fluoxetine delivery

**Figure 7:**
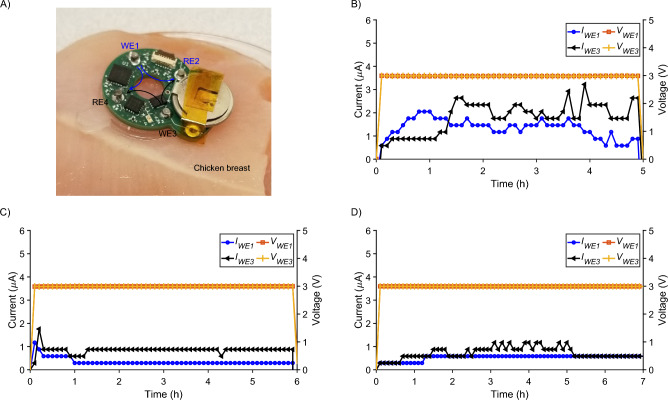
Experimental results for the memory enabled battery-powered system. (**A**) The fabricated battery-powered PCB with memory integrated with PDMS prepared for Flx$^+$ delivery positioned on top of chicken breast. (**B**) WE voltages and currents for Device 1 (5 h of actuation). (**C**) WE voltages and currents for Device 2 (6 h of actuation). (**D**) WE voltages and currents for Device 3 (7 h of actuation).

The battery-powered device with memory was tested to deliver fluoxetine cations (i.e., a charged biomolecule) ex vivo on a chicken breast, as shown in Fig. 7A. To prepare the device, Ag and AgCl wires are used as the WE and the RE, respectively. Since fluoxetine can actually become oxidized under a voltage^22,67^, we prevent any potential changes in delivery or biological effects that could result from this by using Ag as the WE as Ag will oxidize before fluoxetine. All 4 reservoirs are filled with 10 mM Flx HCl solution, and the capillaries are loaded with Flx$^+$. In the WE reservoirs, Flx HCl helps to keep the concentration of Ag$^+$ ions below the toxicity threshold of 30 ppm^68^ (= 2.78 $\times$ 10$^{-4}$ M). This is because Ag$^+$ ions promptly combine with Cl$^-$ ions to form AgCl (solid), which then gets efficiently deposited back to the WE. Moreover, AgCl has a low solubility of 1.3 $\times$ 10$^{-5}$ M that helps to maintain a low concentration of free Ag$^+$ ions ($< 10^{-7}$ M). The hydrogel-filled capillaries serve as an ion exchange barrier, reducing Ag$^+$ ion leakage from the reservoirs compared to a direct contact. This has previously been accepted as a safe option^12,43^. We use a protonated Flx HCl solution (10$^{-2}$ M) which has a pH of 4, so the H$^+$ concentration in the reservoir is 10$^{-4}$ M. The Flx+ ions outnumber the H+ ions by 100:1 and are therefore more likely to be delivered. The hydrogel itself does not have an intrinsic structural property that makes it more selective between H$^+$ and Flx$^+$. Therefore, all the cations present in the reservoir compete with each other to be delivered based on the concentration and diffusion coefficient of the cation^69^. Two pairs of WE and RE [i.e., (WE1, RE2) and (WE3, RE4)] were used to allow for higher total current $I_{WE} = I_{WE1} + I_{WE3}$ and larger coverage of fluoxetine cation delivery over the wound area via the capillary tubes underneath. The applied voltage $V_i$ at each channel for fluoxetine delivery is given in Table 8. The average fluoxetine delivery efficiency is 20±4%, based on HPLC measurements in vitro^22^. The MCU sets the appropriate DAC outputs to either the $V_i = 3$ V (for the WE electrodes) or $V_i = 0$ V (for the RE electrodes) by sending commands to the DAC on the I^2^C bus. Three assembled devices were tested, distinguished by a varied actuation duration of 5, 6, and 7 h. The WE1 and WE3 voltages and currents are measured and stored on the onboard EEPROM, as shown in Fig. 7B, C, and D.

**Table 8:** Applied voltage $V_i$ at the four channels for Flx$^+$ delivery on chicken breast with the memory enabled battery-powered system.

**Channel**	Ch. 1 (WE1)	Ch. 2 (RE2)	Ch. 3 (WE3)	Ch. 4 (RE4)
**Applied voltage** ${{\varvec{V}}}_{{\varvec{i}}}$	3 V	0 V	3 V	0 V

The currents fluctuate with time, likely due to local changes in contact resistance between the capillary tubes and chicken breast induced by substance delivery, variation in device weight distribution on the chicken breast over time, and increasing dryness of the chicken at room temperature and humidity.

## Summary

We demonstrate that the platform’s design allows for the replacement of customizable components while the fabrication provides a repeatable and scalable process, making it a versatile tool for various therapeutic applications. Moreover, we show the potential of the proposed modular wearable bioelectronic system to improve wound healing through H$^+$ delivery in vivo using mouse models. IHC staining shows that the in vivo wounds treated with H$^+$, delivered by the battery-powered device, had an improved M1/M2 ratio compared to control wounds by 35.86%. Additionally, the system’s ability to deliver charged biomolecules continuously for up to 7 h ex vivo demonstrates the potential for long-term treatment delivery.

## Methods

### Ethics declarations: approval for animal experiments

All animal experiments were conducted under the protocol approved by the University of California Davis (UC Davis) Institutional Animal Care and Use Committee (IACUC). All methods were performed in accordance with the UC Davis IACUC guidelines and regulations. All animal studies are reported in accordance with ARRIVE guidelines.

### PCB fabrication and modeling

The PCBs are designed in Autodesk EAGLE. The prototypes are fabricated by a PCB manufacturer. The PCB assembly is done in-house at the Teodorescu lab. Frameless PCB stencils are utilized for solder paste deposition on surface-mount pads. A benchtop reflow oven is used for reflow soldering of surface-mount components. Through-hole components are hand soldered. The 3D models of the PCB are created in Autodesk Fusion 360.

### 3D printing of PDMS molds

The CAD models of the PDMS molds are created in Autodesk Fusion 360. The STL files of the models are imported into the Formlabs PreForm software. The models are optimized for print orientation, layout, and supports before uploading the resulting file to the Formlabs Form 3 SLA printer for printing with Model V2 resin.

### Hydrogel-filled capillary preparation

In order to deliver ions such as H$^+$, the four silica capillaries inserted in each device are filled with a polyanion hydrogel that selectively conducts cations. The hydrogel recipe requires 1 M AMPSA, 0.4 M PEGDA, and 0.05 M photoinitiator (I2959) concentrations to result in a hydrogel with a low swelling ratio (12%) and good conductivity (8.8±0.1 S/m). The AMPSA monomer undergoes a radical polymerization reaction with PEGDA as the crosslinker and I2959 as the photoinitiator. They have all been used in biomedical applications due to their biocompatibility and/or shown to have low levels of cytotoxicity^70–72^. AMPSA offers high cationic conductivity due to the fact that it has a large number of mobile counter ions^73^. PEGDA is non-toxic and safe for biomedical purposes^71^. The structure of hydrogels made of Na-AMPSA and PEGDA has been studied for use as burn dressings^74^. A several-centimeter length of the silica tubing (inner diameter 100 $\mu$m, outer diameter 375 $\mu$m) used for the capillaries is etched with NaOH and further treated with silane A174 to prevent the hydrogel from swelling out of the capillary after UV crosslinking for 5 min at 8 mW/cm$^2$. Following UV curing, the capillary tubes are cut into 5 mm pieces. This designated length allows for the easy insertion of capillaries into the PDMS device, with minimal cutting required to get the capillaries down to the required 2–3 mm final length. Cutting the capillary tubing into small lengths beforehand also allows each tube to be checked for electrical connection and loaded with H$^+$. The capillaries are first soaked in a 0.1 M HCl solution for several hours which diffuses out any unpurified, unreacted AMPSA, I2959, and reaction byproducts, and replaces them with the HCl solution. Both ends of the capillary are then placed in electrolyte wells made from PDMS on a glass slide (see Supplementary Fig. S7 online). 0.5 mm diameter Ag and AgCl wires are used as the WE and RE, respectively, and are inserted at the two ends of each capillary to be loaded. The wells are filled with 0.1 M HCl (source solution) at the WE end and 0.01 M KCl (target solution) at the RE/counter electrode (CE) end. To avoid water splitting, +0.8 V is applied at the WE for 5 min. A clean steady-state current is obtained when there are no hydrogel issues. The typical steady-state current measured when loading the capillaries with H$^+$ is 6 $\mu$A (see Supplementary Fig. S7 online). For fluoxetine delivery, the capillary preparation process is similar where, instead of HCl solution, we use Flx HCl solution for soaking and for loading. The mechanism of conduction within the hydrogel is due to the ionized sulfonate groups provided by the AMPSA monomer, providing selectivity for cation conduction over anions^69^.

### Animal experiments

C57B6 males, wildtype mice (32–32 weeks old, 30–35 g) were used. They were acclimated and supplied with DietGel 93M (ClearH2O) and soaked chow to maintain the body weight for one week before the experiment started. The mice were weighted and shaved 1–3 days prior to the surgery. On the day of surgery (Day 0), the animals were anesthetized with 1–5% isoflurane inhalation, saline and analgesics were injected, and the back skin was prepared with betadine and alcohol washes. One wound was created on the side of the spine by suturing silicon splint rings (16 mm outer and 10 mm inner diameters) on the intended location. A 6 mm biopsy punch generated a full-thickness, excisional wound with the silicon splint to control wound contraction on each mouse. The wounds were covered with a vapor permeable secondary dressing (such as Tegaderm) to secure the devices. For the treatment group, devices deliver H$^+$ continuously for a specified duration to reach a recommended target dose. Daily examination of the wounds, dressings, and device functionality are performed for a total of four days starting on the surgery day (Day 0). On Day 3, the animals were euthanized through cervical dislocation with deep anesthesia to effect and the wound tissue are harvested for IHC staining.

### Histology

At the end of each experiment, after euthanizing the mice, wounds were excised and placed into a paraformaldehyde solution to fix for 24 h. Next, the fixed tissues were processed in a tissue processor for FFPE tissue histology. During processing, the tissues were dehydrated and impregnated with paraffin wax to preserve the tissue structure. Processed tissues were then embedded into paraffin blocks and cut into 5 $\mu$m thick sections using a microtome, and the sections were placed onto glass slides. After some drying time, these sections were used for IHC staining to determine the M1/M2 ratio.

### Macrophage IHC staining

We stained both M1 and M2 macrophages to get an accurate cell count. 4’,6-diamidino-2-phenylindole (DAPI) is used to mark the nuclei, F4/80 is used to show all macrophages, and iNOS is used to stain M1 while CD206 is used to stain M2. Only those cell nuclei (labeled with DAPI) that overlapped/surrounded by both markers were considered as macrophages, i.e., M1 (iNOS + F4/80 + DAPI) and M2 (CD206 + F4/80 + DAPI). The IHC staining of formalin-fixed, paraffin-embedded tissue sections were achieved over two days as follows:

- Day 1
  1. Deparaffinize slides: Place slides in 2 washes of Xylene for 10 min each, 1 wash of Xylene/ETOH for 4 min, 2 washes of 100% EtOH, 2 washes of 95% EtOH, 2 washes of 90% EtOH,1 wash of 70% EtOH, and 1 wash of 50 % ETOH for 4 min per wash. Pre-warm Sodium Citrate pH 6.0 in the rice cooker after the Xylene washes.
  2. Wash/tilt slides 3$\times$ in 1$\times$ PBS (or TBS) + 0.1% Tween-20 for 5 min each time.
  3. Steam slides in Sodium Citrate Buffer (pH 6.0) + 0.05% Tween-20 inside of rice cooker for 30 min.
  4. Cool slides to room temp in 1x PBS (or TBS) or H2O for 10-15 min (keep wet).
  5. Wash/tilt slides 2$\times$ 5 min in 1$\times$ PBS (or TBS) + 0.1% Tween.
  6. Use a PAP/Immuno-pen to outline a hydrophobic barrier for incubating tissue.
  7. Apply Permeabilization/Blocking Buffer for 2 h in a humidified slide box.
  8. Apply primary antibody diluted in Antibody dilution buffer and incubate overnight at 4 $^{\circ }$C in a humidified chamber with primary antibodies: Rat anti-F4/80 (dilution 1:50; MCA497G, BIO-RAD, Hercules, CA), Rabbit anti-iNOS (dilution 1:100; PA3-030A, Thermo Fisher Scientific) and Goat anti-CD206 (dilution 1:100; PA5-46994, Thermo Fisher Scientific).
- Day 2
  1. Wash the slides 3$\times$ in 1$\times$ PBS or TBS (+ 0.1% Tween) for 5 min each.
  2. In the dark, apply corresponding Alexa Fluor-conjugated secondary antibodies: Donkey Anti rat-AlexaFluor 488, Donkey Anti rabbit-AlexaFluor 647, and Donkey Anti goat-AlexaFluor 568, dilution 1:200, Thermo Fisher Scientific), for sections from step 8 (on Day 1), and incubate for 1 h and 30 min. During the incubation time, make sure slides are light protected from this step.
  3. Wash/tilt 3$\times$5 min in 1$\times$ PBS/TBS (+ 0.1% Tween).
  4. Dilute sufficient DAPI stock by 1:1000 in DI H2O and place on slides for 10 min.
  5. Wash/tilt 3$\times$5 min in 1$\times$ PBS or TBS.
  6. Drain excess PBS using paper towels, while carefully avoiding tissue.
  7. Apply ~22.5 $\mu$L of anti-fade mounting media (SlowFade Mountant; S36936, Thermo Fisher Scientific) and gently add cover slips over sections to carefully not make bubbles. Use tweezers to guide the coverslip slowly.
  8. Put nail polish around the edges and let dry for 15 min overnight.

Fluorescence images were acquired using a Keyence automated high-resolution microscope (BZ-X800, KEYENCE, Itasca, IL). The images were processed using ImageJ and CellProfiler 4.2 software.

### Supplementary Information

Supplementary Information 1.

## Data Availability

The data generated and analyzed during this study are available from the corresponding author on reasonable request.
