# Supplementary material for: A system for bioelectronic delivery of treatment directed toward wound healing
Source: Sci Rep. 2023 Sep 7;13:14766. doi: 10.1038/s41598-023-41572-w (PMC10485133; doi:10.1038/s41598-023-41572-w)
Supplement: Supplementary file 1 — Supplementary Information 1. [file 41598_2023_41572_MOESM1_ESM.pdf]

Supplementary Information

A system for bioelectronic delivery of treatment directed toward wound healing

Prabhat Baniya[1,*], Maryam Tebyani[1,5], Narges Asefifeyzabadi[1], Tiffany Nguyen[1], Cristian Hernandez[1], Kan Zhu[2,3], Houpu Li[1], John Selberg[1], Hao-Chieh Hsieh[1], Pattawong Pansodtee[1,5], Hsin-ya Yang[2], Cynthia Recendez[2,3], Gordon Keller[1], Wan Shen Hee[1], Elham Aslankoohi[1], Roslyn Rivkah Isseroff[2], Min Zhao[2,3], Marcella Gomez[4], Marco Rolandi[1,*], and Mircea Teodorescu[1,5,*]

[1]Department of Electrical and Computer Engineering, University of California Santa Cruz, Santa Cruz, California, 95064, USA
[2]Department of Dermatology, School of Medicine, University of California Davis, Sacramento, California, 95816, USA
[3]Department of Ophthalmology & Vision Science, University of California Davis, Sacramento, California, 95817, USA
[4]Department of Applied Mathematics, University of California Santa Cruz, Santa Cruz, California, 95064, USA
[5]Genomics Institute, University of California Santa Cruz, Santa Cruz, California, 95060, USA
[*]pbaniya@ucsc.edu, mrolandi@ucsc.edu, mteodore@ucsc.edu

Supplementary table, figures, and text

Biocompatibility evaluation

The study's objective was to evaluate the biocompatibility of materials of the portion of our bioelectronic devices that come in contact with the wound. The devices were placed subcutaneously in rabbit models by trained experts from DaVINCI Biomedical Research Products, Inc. The materials, PDMS + Parylene-C, PDMS + Parylene-C + Capillary Tube, and PDMS + Parylene-C + Capillary Tube + Hydrogel, selected to be the testing samples can be seen on the study design Table S1. The study population consisted of 5 animals: 2 males and 3 females. There was 1 control group and 2 test groups. Each animal was scheduled to receive 12 implants: 6 on the right and six 6 on the left dorsal region. Each animal was implanted according to the study design Table S1. After completion of the survival period, the animals were euthanized; all implant sites were harvested, preserved in 10% neutral buffered formalin (NBF), and referred for histopathology. The results indicate there were no adverse events in any of the 5 animals. The study objectives were met. The rabbits did not exhibit any test article-related abnormalities throughout the study. All 5 animals successfully underwent the surgical procedures, recovered, and completed the 29-day survival period. At the completion of the study, all animals were evaluated as being in optimum health.

Group ID	Animal ID/ Sex	Testing samples	Biocompatibility evaluation
1	1F	Control Article: PDMS + Parylene-C	The ISO 10993-6:2016 Ranked Reactivity scores for Test Article 1 and Test Article 2 implants relative to the Control Article implant were classified as minimal to no reaction in the subcutaneous rabbit model at 29 days. No pathologic changes indicative of toxic injury were found in the non-target organs evaluated in any of the 5 animals.
2	5M<br>2F	Test Article 1: PDMS + Parylene-C + Capillary Tube<br>Test Article 1: PDMS + Parylene-C + Capillary Tube	
3	6M<br>3F	Test Article 2: PDMS + Parylene-C + Capillary Tube + Hydrogel<br>Test Article 2: PDMS + Parylene-C + Capillary Tube + Hydrogel	

Table S1. Biocompatibility evaluation of the portion of our bioelectronic devices that come in contact with the wound: PDMS, Parylene-C, capillary tube, and hydrogel.

Wired and battery-powered bioelectronic devices

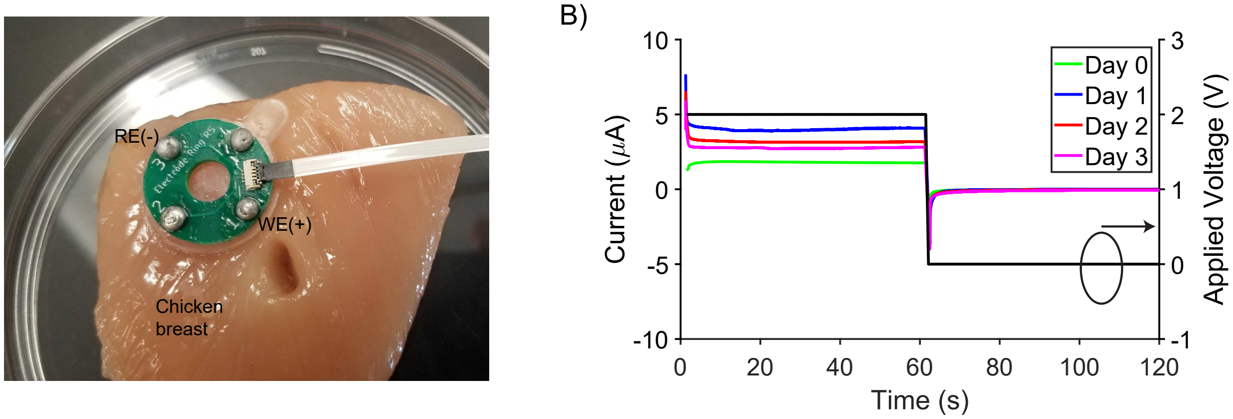

Figure S1. A) Wired device placed on the back of a chicken breast for short duration H^+ delivery ex vivo, using an external voltage controller. B) Current response on Days 0, 1, 2, and 3, where 2 V was applied across WE and RE for 60 s, followed by 0 V application.

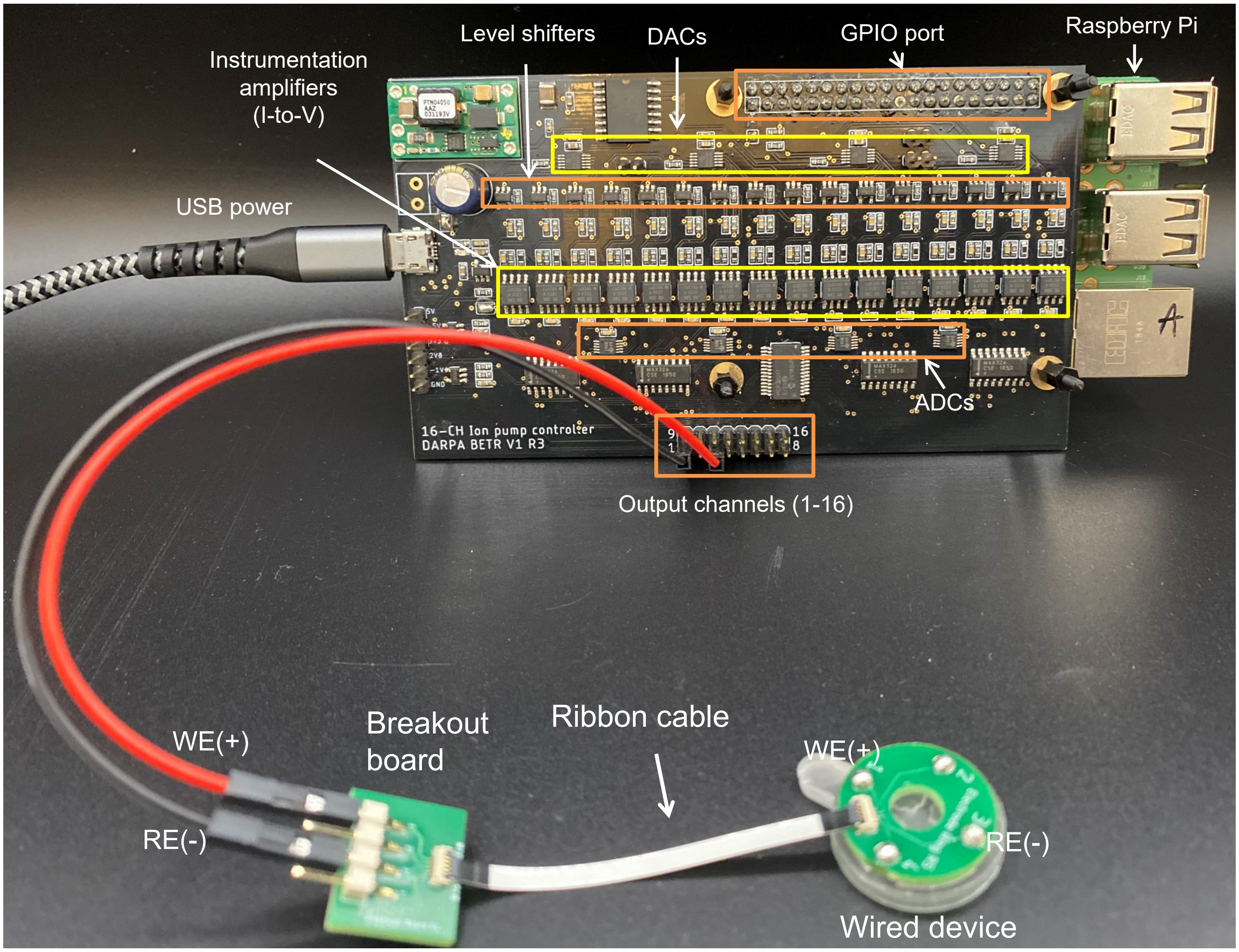

Figure S2. The external voltage controller connected to the wired device. The controller is a PCB attached on top of a Raspberry Pi 3B+. All the major electronic components are highlighted in boxes.

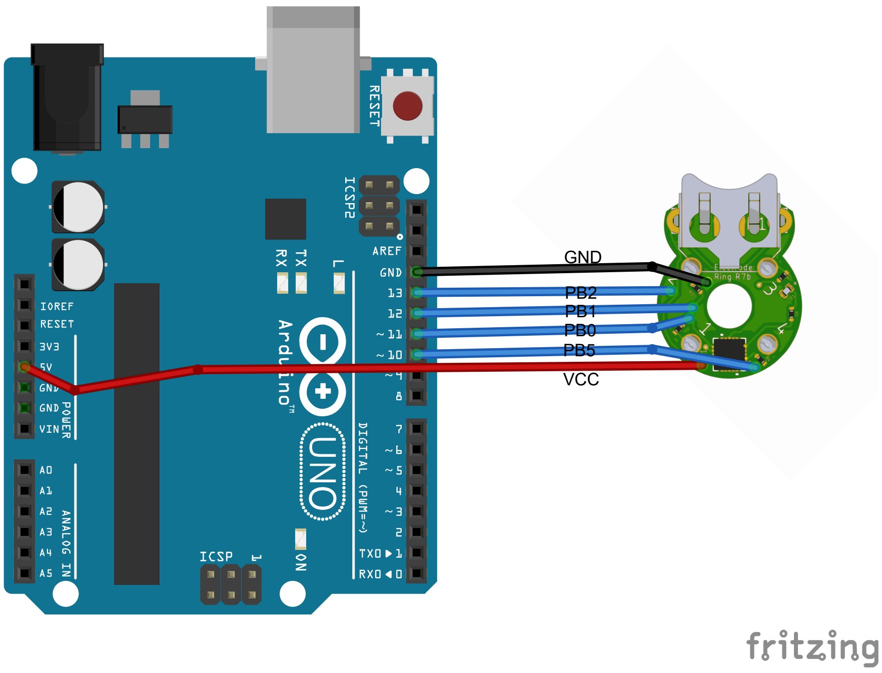

Figure S3. For programming the onboard ATtiny85V, its pins PB5, PB0, PB1, PB2, VCC, and GND (accessible through the programming vias on the PCB) are respectively wired to the four SPI pins (10, 11, 12, 13), 5V, and GND of the Arduino Uno. The 3 V coin battery must be removed when programming. This image was created with Fritzing. After the MCU is flashed, the reset pin (PB5) is disabled by writing 0x5F to the hfuse by runnning the AVRdude.exe program of the Arduino IDE from the Windows' command line. This prevented the MCU from unintentionally resetting during handling and testing.

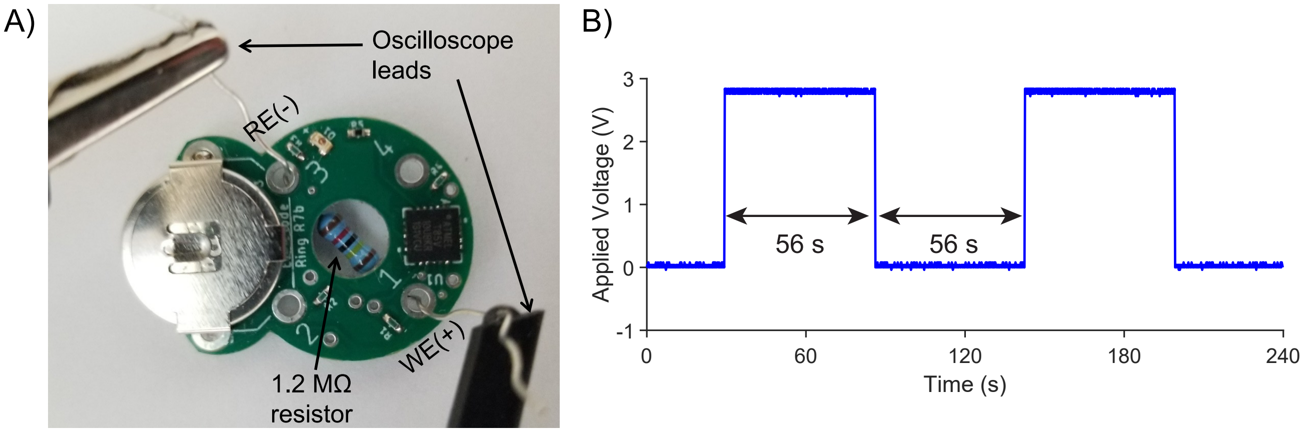

Figure S4. A) Experimental setup to verify output voltage levels under load on the battery-powered controller. B) Measured voltage and timing on a digital oscilloscope.

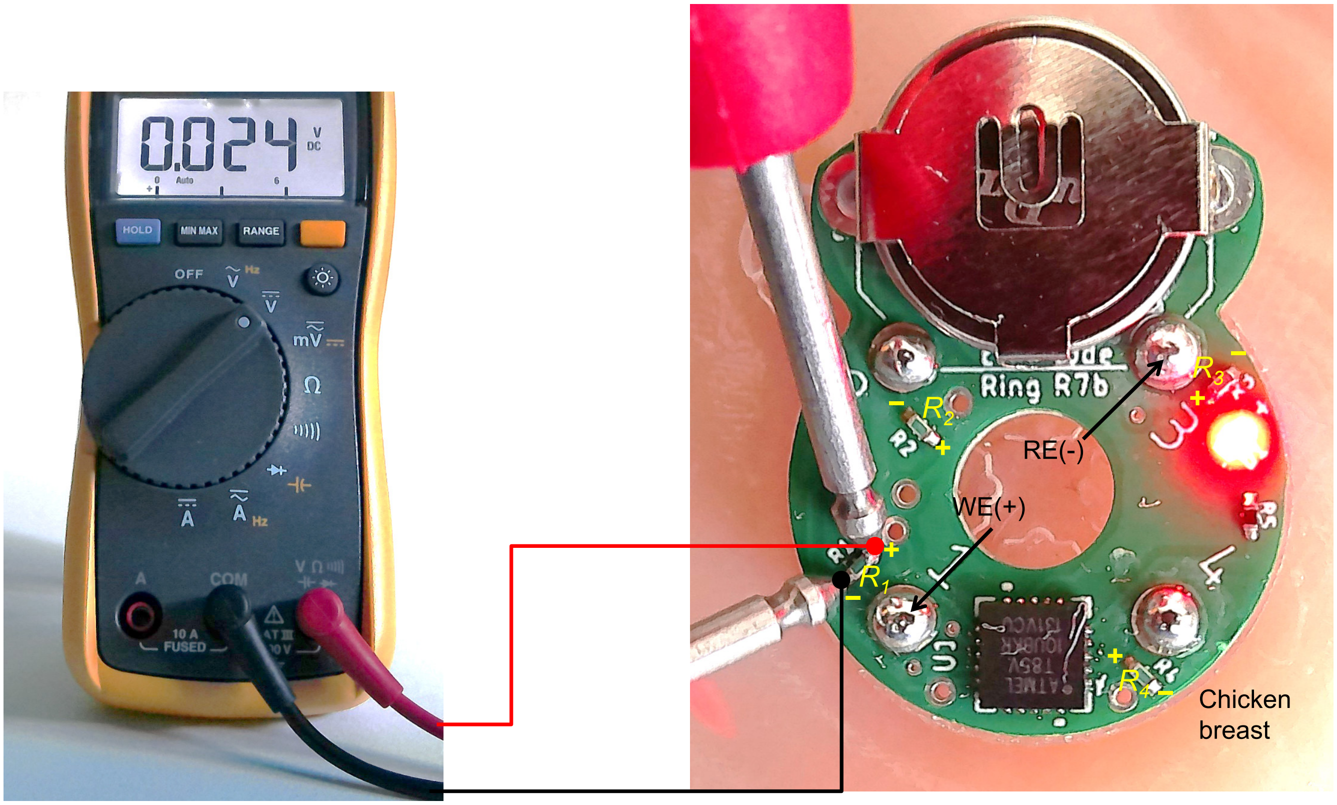

Figure S5. Experimental setup to measure current with battery-powered device which is placed on the back of a chicken breast for long duration H^+ delivery ex vivo. Current at each channel can be measured in μA by first measuring the voltage (in Volts V) across a sense resistor (R_i = 10 kΩ) using a multimeter and multiplying that number by 100. In this demonstration, the multimeter is showing 0.024 V drop across R_1 which means that 2.4 μA of current is flowing at channel 1 when 3 V is applied across WE and RE.

M1/M2 macrophage and IHC staining

Macrophages, among the first cell types to traffic to the wound, fulfill many critical roles throughout wound healing. They form an immune defense line, promote and resolve inflammation, remove dead cells and cell debris, and support cell proliferation and tissue restructure. These tasks are accomplished with their plasticity for change into multiple functional phenotypes. The best documented phenotypic change is the in vitro shift from the M1 macrophage inflammatory phenotype (classically activated) to the M2 (alternatively activated) anti-inflammatory pro-reparative phenotype. M1 is characterized by secretion of IL-1, TNFa, IL-6, IL-12, MMPs, and other cytokines; M2 is characterized by production of arginase, TGFb, CCL18, PGE2, and IL-10, and up-regulation of scavenger receptors (CD206, CD163). This shift is highly dependent on the signals in the microenvironment, and thus, the in vivo phenomenon may present as a continuum rather than a bipolar paradigm. Because of these distinct roles, macrophages undergo a precisely regulated dynamic transition in their function in time and space.

M1 and M2 macrophages were manually counted based on the double-positive staining with blind evaluation. For each mouse, five adjacent regions at the wound center were imaged at 40× magnification for macrophage quantification, as shown in Figure S6A. A typical set of stained tissue slices (at wound centers) gathered from a control and an H^+ – treated mouse is shown in Figure S6B. All data in Figure S6C are presented as mean ± standard deviation. On average, the inflammatory phase is shortened in the treatment group as measured by the decrease in the M1/M2 ratio.

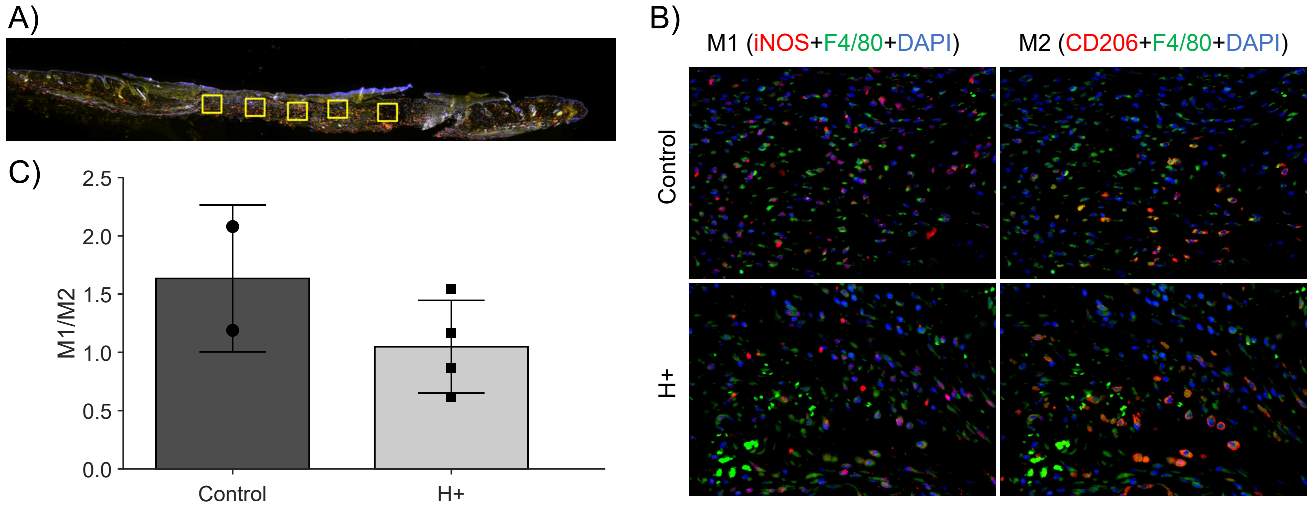

Figure S6. IHC staining results from the four-day in vivo experiments using the battery-powered system. A) IHC staining on a tissue sample where five regions (yellow squares) were sampled. B) Representative M1 and M2 macrophage staining at wound centers from control and H^+ – treated mice. C) Plot of the M1/M2 ratio data with calculated average and standard deviation displayed for the control and H^+ – treated wounds. On average, M1/M2 ratio of the H^+ – treated wounds is 35.86% lower compared to the control wound.

Hydrogel-filled capillary loading

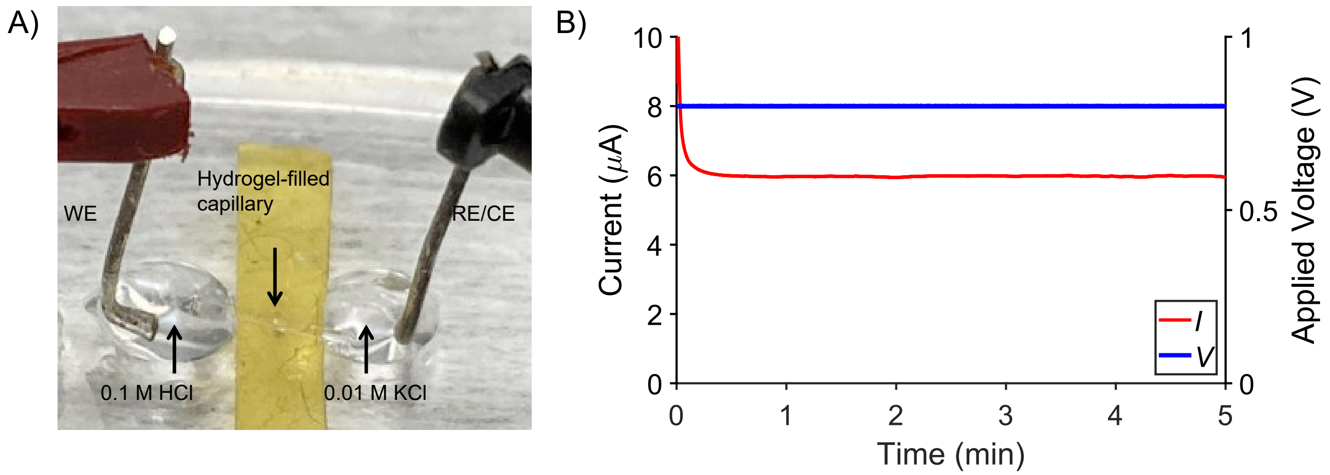

Figure S7. A) Experimental setup and current response for capillary loading with H^+. A) A 0.5 mm diameter Ag wire is used as the WE and a 0.5 mm AgCl wire is used as the CE/RE to load a 5 mm long hydrogel-filled capillary. B) Typical current response when 0.8 V is applied for five minutes to load H^+. The steady-state current is around 6 μA.
